# Differential Expression of Na^+^/H^+^-Exchanger (NHE-1, 2, and 4) Proteins and mRNA in Rodent's Uterus under Sex Steroid Effect and at Different Phases of the Oestrous Cycle

**DOI:** 10.1155/2013/840121

**Published:** 2013-02-11

**Authors:** Khadijeh Gholami, Sekaran Muniandy, Naguib Salleh

**Affiliations:** ^1^Department of Molecular Medicine, Faculty of Medicine, University of Malaya, 50603 Kuala Lumpur, Malaysia; ^2^Department of Physiology, Faculty of Medicine, University of Malaya, 50603 Kuala Lumpur, Malaysia

## Abstract

Precise uterine fluid pH regulation may involve the Na^+^/H^+^-exchanger (NHE). We hypothesized that NHE isoforms are differentially expressed under different sex steroid treatment and at different oestrous cycle phases which may explain the uterine fluid pH changes observed under these conditions. *Method*. Oestrous cycle phases of intact WKY rats were identified by vaginal smear. Another group of rats was ovariectomized and treated with 0.2 **μ**g 17**β**-oestradiol (E), 4 mg progesterone (P), and E followed by P (E + P). The animals were then sacrificed and the uteri were removed for mRNA and protein expression analyses by real-time PCR and western blotting, respectively. NHE isoforms distribution was detected by immunohistochemistry (IHC). *Results*. NHE-1 mRNA and protein were upregulated at diestrus (Ds) and following P treatment. Meanwhile, NHE-2 and NHE-4 proteins and mRNA were upregulated at proestrus (Ps) and estrus (Es) and following E treatment. NHE-1 was found predominantly at the apical membrane, while NHE-2 and NHE-4 were found at the apical and basolateral membranes of the luminal epithelia. NHE-4 is the main isoform upregulated by E. *Conclusion*. Differential expressions of uterine NHE isoforms 1, 2, and 4 could explain the observed changes in the uterine fluid pH under these conditions.

## 1. Introduction

Sodium hydrogen exchanger (NHE) with a stoichiometry of 1 H^+^: 1 Na^+^ is an integral plasma membrane protein and is typically involved in the exchange of intracellular H^+^ with external Na^+^, according to the concentration gradient [1]. NHE belongs to a solute carrier family 9 (SLC9) and consists of nine members (NHE-1–9) that are present in almost all living cells [2, 3]. NHE is involved in cell volume changes and intracellular pH regulations as well as acid-base balance [4, 5]. Its activity also facilitates cellular adhesion, migration, and proliferation [6]. NHE plays an important role in many absorptive and secretory epithelia. It is one of a major nonnutritive Na^+^ absorptive pathway in the intestine and kidney [4]. In addition to H^+^ exchanged, NHE is indirectly involved in about 15% of HCO_3_^−^ reabsorption from the thick ascending loop of Henle [7]. In the epididymis, NHE-2 and NHE3 contribute to Na^+^ reabsorption in exchanged with H^+^ which resulted in luminal fluid acidification [8].

Nearly a decade ago, Wang et al. [5] detected the presence of NHE isoform 1, 2, and 4 mRNAs in the endometrial cells isolated from immature mice uterus. Since then, no follow-up studies have been performed to investigate the cyclical changes in the expression of these isoforms in the adult uterus throughout the oestrous cycle phases or under different sex steroid influence. While NHE involvement in the luminal fluid pH regulation has been reported in many tissues [5, 7, 8], there is currently a lack of information on its role in the uterine fluid pH changes. An understanding of the mechanisms underlying this is important as it has an implication towards a normal fertility. A relatively high pH has been documented under E effect [9] and at around the time of ovulation [10], coincides with a high HCO_3_^−^ content. Meanwhile, our unpublished observation indicates that uterine fluid pH is significantly reduced at Ds when the level of endogenous P is high. In addition to regulating the changes in the luminal fluid pH, in tissue such as epididymis, NHE has also been reported to be involved in H_2_O reabsorption secondary to Na^+^ reabsorption, which is associated with luminal fluid acidification [5].

We hypothesized that the changes in the uterine fluid pH as observed throughout the oestrous cycle phases are related to differential expressions of NHE isoforms under different hormonal conditions. Since the level of E and P fluctuates throughout the oestrous cycle, there is a possibility that these hormones can affect uterine NHE expression. In view of this, the aim of our study is to investigate the changes in uterine NHE isoforms expression under different effects of sex steroids and at different phases of the oestrous cycle in order to provide the molecular basis underlying these observed uterine fluid pH changes.

## 2. Materials and Methods

### 2.1. Animals and Hormones Treatment

Wistar-Kyoto (WKY) rats of approximately 2 months of age, weighting 200–250 g, were maintained under normal conditions of lighting (lights on from 06:00 to 18:00 hr) and room temperature (±24°C) and were fed on soy-free diet (Gold Coin Pellet) and tap water *ad libitum*. All experimental procedures were approved by the Faculty of Medicine Animal Care and Use Committee (ACUC), UM, with the ethics number 14/9/2009/FIS/NS. E and P were purchased from Sigma-Aldrich (UK). All drugs were dissolved in peanut oil. Ten days after ovariectomy, the rats, divided into 7 groups of 6 rats per group, received the following drug treatment: 0.2, 2, 20, and 50 *μ*g/mL E each for three consecutive days, 4 mg/mL P for three consecutive days, 0.2 *μ*g/mL E for three consecutive days followed by 4 mg/mL P for another 3 days (E + P), and three days treatment with peanut oil which acts as a control. The drugs were administered via a subcutaneous injection behind the neck cuff.

### 2.2. Oestrous Phases Identification

Vaginal secretion was collected with a plastic pipette filled with 10 *μ*L of normal saline (NaCl 0.9%). The tip of the pipette was inserted into the rat vagina, but not deeply to avoid cervical stimulation. The saline was then flushed into the vagina and immediately recovered and the unstained materials collected were placed onto a glass slide and which was then observed under a light microscope. The proportions among different cells were used to determine the oestrous cycle phases according to Marcondes et al. [11]. Following steroid treatment or oestrous phases identification, the rats were sacrificed by cervical dislocation. Uterine tissues were then removed for protein and mRNA studies.

### 2.3. Protein Expression Analysis by Western Blotting

Snap-frozen tissues were homogenized using a sonicator with PRO-PREP (Intron) extraction solution in the presence of protease inhibitors. The total cell protein was obtained by centrifugation at 13000 g for 15 minutes at 4°C. Proteins were then quantified by Micro BCA Protein Assay Kit from Thermo Scientific (UK). The samples were boiled for 5 minutes and were then subjected to 12% SDS-PAGE. Proteins were transferred onto PVDF membranes (BIORAD) at a constant voltage. The membranes were incubated with primary antibody (goat) for NHE-1, NHE-2, and NHE-4 (1 : 1000) and were then incubated with secondary antibody (anti-goat) conjugated to a horseradish peroxidase (HRP) (1 : 2000). The membrane was then visualized by Opti-4CN Substrate Kit from BIORAD. *β*-actin (abcam, UK) was used as a loading control. The photos of the blots were captured with a gel documentation system and the density of each band was determined by using Image J software. The ratio of each band/*β* actin was considered as the expression level of the target proteins. All NHEs antibodies were purchased from Santa Cruz (USA).

### 2.4. mRNA Expression Analysis by Real-Time PCR (qPCR)

RNALater was used to maintain the RNA integrity prior to extraction. Total RNA was extracted from the uterine tissue using Ribo pure kit from Ambion (USA) according to the manufacturers' instruction, with TRI reagent. RNA concentration and purity was assessed by 260/280 UV absorption ratios (Gene Quant 1300, UK). RNA integrity was evaluated by gel electrophoresis. Samples were then aliquots for each run, to avoid freeze-thaw cycle. One step real-time PCR was used to evaluate gene expression, with the application of TaqMan RNA-to-CT 1-Step Kit (Applied Biosystems, USA). Total volume of 10 *μ*L per reaction was used. Reaction material include: 5 *μ*L master mix, 0.225 *μ*L reverse transcriptase, 3.25 *μ*L water and 1 *μ*L sample that contained 100 ng total RNA. Hprt1 and GAPDH were used as reference genes. All experiments were done in 3 biological replicates. PCR program includes 15 minutes, 48°C reverse transcriptase, 10 minutes 95°C activation of ampli-Taq gold DNA polymerase, denature at 95°C for 15 seconds and annealing at 60°C for 1 minutes. Denaturing and annealing was performed for 40 cycles. All TaqMan (hydrolysis probe) assays were purchased from Applied Biosystem (USA), assay ID for NHE-1, -2 and -4 was Rn01418250_m1, Rn00688610_m1 and Rn01437220_m1, respectively. Data assist v3 from Applied Biosystems and SPSS 13 were used for data analysis. 

### 2.5. Protein Localization by Immunohistochemistry (IHC)

Uteri obtained from WKY rats were immediately transferred into 4% paraformaldehyde and, after tissue processing, were embedded into the paraffin wax. Paraffin sections of 5 *μ*m were dewaxed and hydrated. Antigen retrieval was performed using trisodium citrate buffer for 10 minutes in a microwave oven. After washing with PBS, the section was incubated in PBS containing 10% (v/v) H_2_O_2_ for 30 minutes to quench the activity of the endogenous peroxidase. Nonspecific binding was blocked by incubation in 5% BSA (Innovative, USA), and the sections were then incubated with primary antibody for NHE-1, -2, and -4 (1 : 50) for 90 minutes. The tissues were then incubated with goat biotinylated secondary antibody (1 : 200) for 30 minutes at room temperature. This was followed by incubation in Avidin and biotinylated HRP in PBS for 30 minutes. Finally, the antigens were visualized using DAB (Diaminobenzidine HCl) staining, which gave dark-brown stained at the sites of antibody binding. The sections were then counterstained with hematoxylin for nuclear staining. All reagents for IHC were purchased from Santa Cruz (USA).

### 2.6. Evaluation of Immunostaining

The relative intensity of the products of immunoreaction at the apical and basolateral membranes of the epithelial cells of the uterine sections were evaluated and graded blindly by three independent observers by using a light microscope (Olympus, Japan) at 100x magnification. The staining intensity was estimated semi-quantitatively on a scale of 0–3+ (+++) as the following: −, no detectable stain; −/+, faint; +, moderate; ++, strong; and +++, very intense staining, as previously described [12].

### 2.7. Statistical Analysis

For real-time PCR, western blotting and IHC, 6 rats per treatment groups were used. The density of protein bands in western blotting was analyzed by Image J software, and the results were presented as a ratio of target protein to *β*-actin. One way Anova, with Tukey's post hoc test, was used to check for the level of significance with *P* value of less than 0.05 was considered as significant.

## 3. Results

### 3.1. Analysis of NHE-1, NHE-2, and NHE-4 mRNA Expression

**Figure 1:**
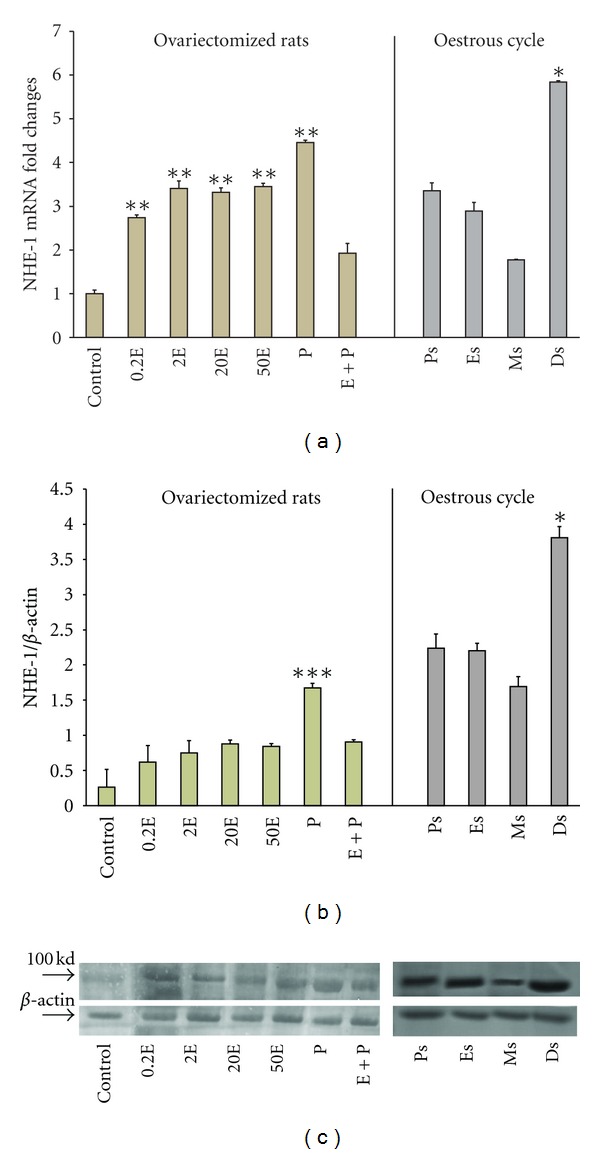
Real-time PCR (a) and Western blot analysis (b) of the total uterine homogenate; representative image of Western blots (c) of NHE1 in steroid replaced ovariectomized rats and in rats at different phases of the oestrous cycle. Results indicate that NHE1 mRNA and protein expressions were increased following P treatment and at Ds stage of the oestrous cycle. Control: peanut oil, 0.2E: 0.2 *μ*g 17*β*-oestradiol, 2E: 2 *μ*g 17*β*-oestradiol, 20E: 20 *μ*g 17*β*-oestradiol, 50E: 50 *μ*g 17*β*-oestradiol, P: progesterone, E + P: 0.2 *μ*g 17*β*-oestradiol + progesterone. Ps: proestrus, Es: estrus, Ms: metestrus, Ds: diestrus.: *P < 0.05, **P < 0.01, ***P < 0.001. *n* = 6 rats per group.

In Figure 1(a), NHE-1 mRNA expression was the highest following P treatment (4.5 folds increased). There was a dose-dependent increase in the mRNA level with increasing doses of E (2.8 to 3.5 folds increased). Treatment with E followed by P resulted in a significant inhibition in mRNA expression to approximately 1.95 fold, which was more than two times lesser than in the P treated group. The significant of this finding was however unknown. Meanwhile, throughout the oestrous cycle, NHE-1 mRNA expression was the highest at Ds (5.9 folds increased), followed by Ps and Es (3.4 and 2.9 folds increased, resp.). These findings suggested that NHE-1 mRNA expression was upregulated under P dominance.

**Figure 2:**
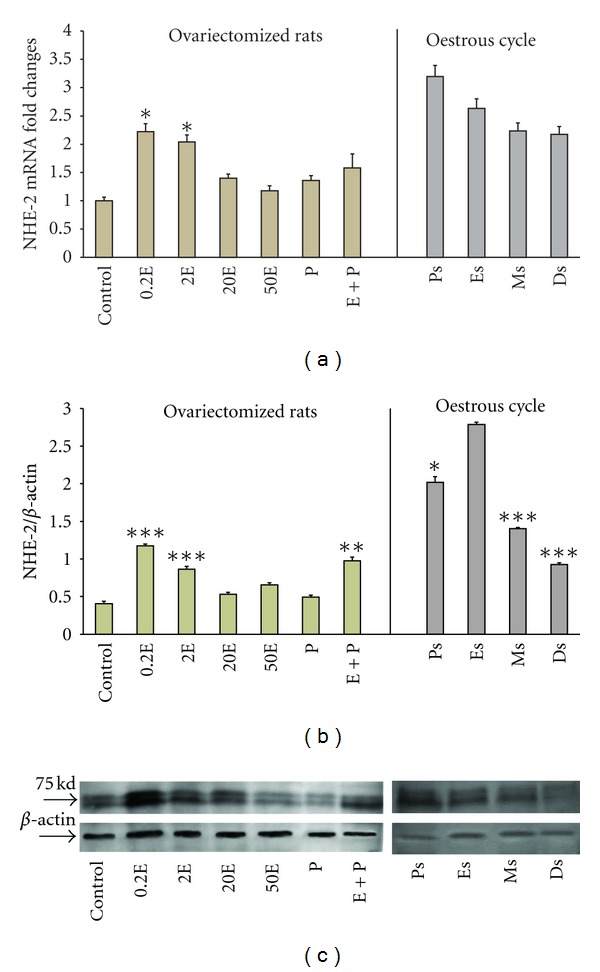
Real-time PCR (a) and Western blot analysis (b) of the total uterine homogenate; representative image of Western blots (c) of NHE2 in steroid replaced ovariectomized rats and in rats at different phases of the oestrous cycle. Results indicate that NHE2 mRNA and protein expressions were increased following E treatment and at Ps and Es stages of the oestrous cycle. Control: peanut oil, 0.2E: 0.2 *μ*g 17*β*-oestradiol, 2E: 2 *μ*g 17*β*-oestradiol, 20E: 20 *μ*g 17*β*-oestradiol, 50E: 50 *μ*g 17*β*-oestradiol, P: progesterone, E + P: 0.2 *μ*g 17*β*-oestradiol + progesterone. Ps: proestrus, Es: estrus, Ms: metestrus, Ds: diestrus.: *P < 0.05, **P < 0.01, ***P < 0.001. *n* = 6 rats per group.

In Figure 2(a), NHE-2 mRNA expression was the highest in the group receiving 0.2E with a 2.3-fold increased. Treatment with increasing doses of E resulted in a reduction in the mRNA expressions (2.05-fold and 1.2-fold reduction with 2E and 50E, resp.). P treatment however resulted in a reduced expression of NHE-2 mRNA (1.9-fold). Although there was a slight increase in the mRNA level in the E + P group, this was, however, not significantly differ from the P-treated group. Changes in NHE-2 mRNA level throughout the oestrous cycle were consistent with the changes following steroids treatment. At Ps, NHE-2 mRNA expression was the highest with a 3.2 folds increased which coincide with a high level of endogenous E, however at Es, there was only a 2.6 folds increased. The lowest mRNA expression was noted at Ds when the circulating level of P was high (2.1-fold increased).

**Figure 3:**
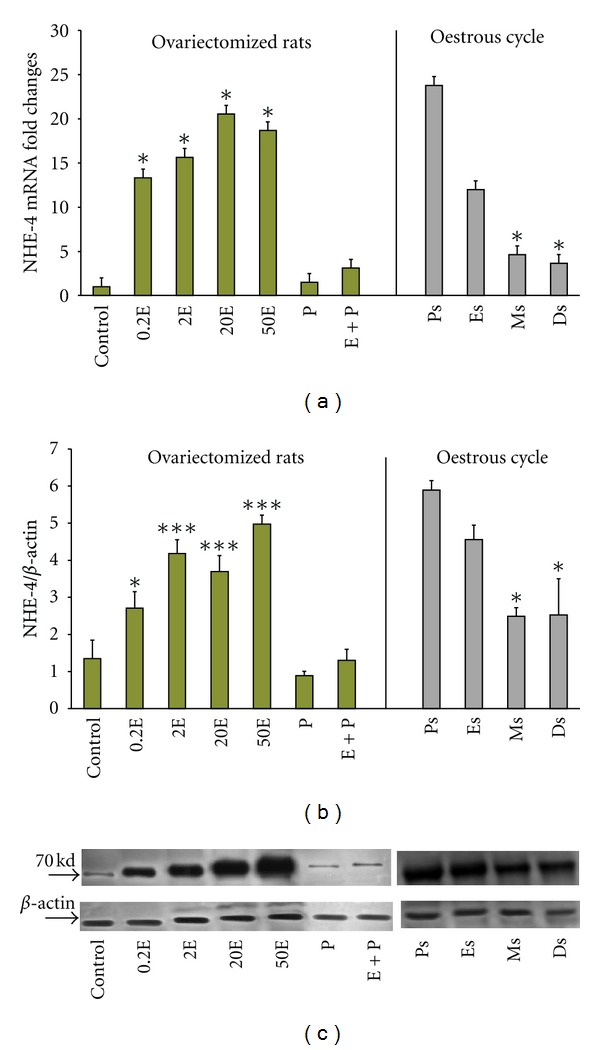
Real-time PCR (a) and Western blot analysis (b) of the total uterine homogenate; representative image of Western blots (c) of NHE4 in steroid replaced ovariectomized rats and in rats at different phases of the oestrous cycle. Results indicate that NHE4 mRNA and protein expression were increased following E treatment and at Ps and Es stages of the oestrous cycle. NHE4 is the most abundant NHE isoform expressed in the uterus under E influence. Control: peanut oil, 0.2E: 0.2 **μ**g 17*β*-oestradiol, 2E: 2 **μ**g 17*β*-oestradiol, 20E: 20 **μ**g 17*β*-oestradiol, 50E: 50 **μ**g 17*β*-oestradiol, P: progesterone, E + P: 0.2 **μ**g 17*β*-oestradiol + progesterone. Ps: proestrus, Es: estrus, Ms: metestrus, Ds: diestrus.: *P < 0.05, **P < 0.01, ***P < 0.001. *n* = 6 rats per group.

In Figure 3(a), NHE-4 mRNA expression was the highest following treatment with E with 14-fold increased following 0.2E treatment and 20 folds increased following treatment with 20E. P treatment resulted in a significant inhibition in the mRNA expression with only a 1.5-fold increase. Treatment with E followed by P resulted in a significant reduction in NHE-4 mRNA expression (3.0-fold) as compared to 0.2E. Meanwhile, throughout the oestrous cycle, NHE-4 mRNA expression was the highest at Ps (24-fold increased), followed by Es (12-fold increased), which were consistent with a high endogenous E. At metestrus (Ms) and Ds however, the levels were significantly reduced with 4.9 and 4.0 folds increased respectively. The expression of NHE-4 mRNA at Ds was 6 times lesser than at Ps, suggesting that a high endogenous P inhibits NHE-4 mRNA expression.

In general under E influence, NHE-4 was the most abundant uterine NHE isoform. The expression of NHE-4 mRNA exceeds NHE-2 mRNA by nearly 7-fold. Under P dominance, however, there was only a slight increase in the NHE-1 mRNA expression as compared to E dominance.

### 3.2. Analysis of NHE-1, NHE-2, and NHE-4 Protein Expression

In Figure 1(b), NHE-1 protein expression was significantly increased following treatment with P (1.7-fold). E treatment resulted in a dose-dependent increased in NHE-1 protein expression (0.6–0.9-fold). In the E + P group, the amount of protein expressed was significantly lower than in the P treated group. The significance of this finding was unknown. Meanwhile, NHE-1 protein was expressed the highest at Ds (3.7-fold increased) than at other stages of the oestrous cycle (2.2- and 2.15-fold increased at Ps and Es, resp.). The lowest expression was noted at Ms (1.6-fold). The molecular weight of NHE-1 was observed at 100 kDa (Figure 1(c)).

In Figure 2(b), the expression of NHE-2 protein was the highest in the 0.2E-treated group. Increasing E doses resulted in a parallel decreased in the amount of NHE-2 protein expressed. P treatment resulted in a significantly lower expression (0.45-fold) as compared to 0.2E. Treatment with E + P resulted in a significant increase in NHE-2 protein expression as compared to P treatment alone. Meanwhile, throughout the oestrous cycle, the expression was the highest at Es (2.75-fold) and the lowest at Ds (0.95-fold) which was consistent with a high endogenous E and a high endogenous P, respectively. The molecular weight of NHE-2 was observed at 75 kDa (Figure 2(c)).

In Figure 3(b), NHE-4 protein was expressed the highest following E treatment. There was a dose-dependent increased in the level of NHE-4 protein with increasing E doses (2.8-fold and 5.0-fold increased with 0.2E and 50E resp.). P treatment however resulted in a significant inhibition of NHE-4 protein expression in which there was only a 0.9 fold increased. Treatment with E + P did not cause any significant changes in the expression as compared to P treatment alone. Meanwhile, throughout the oestrous cycle, NHE-4 protein was expressed the highest at Ps (6.0-fold) and Es (4.65-fold). The lowest expression was noted at Ms and Ds (2.5- and 2.6-fold, resp.). The molecular weight of NHE-4 was observed at 70 kDa (Figure 3(c)).

In general, NHE-4 protein was the main isoform expressed under E dominance although the expression of NHE-2 protein was also increased. Meanwhile, NHE-1 protein expression was slightly increased under P as compared to E.

### 3.3. Analysis of NHE-1, NHE-2, and NHE-4 Protein Distribution

**Figure 4:**
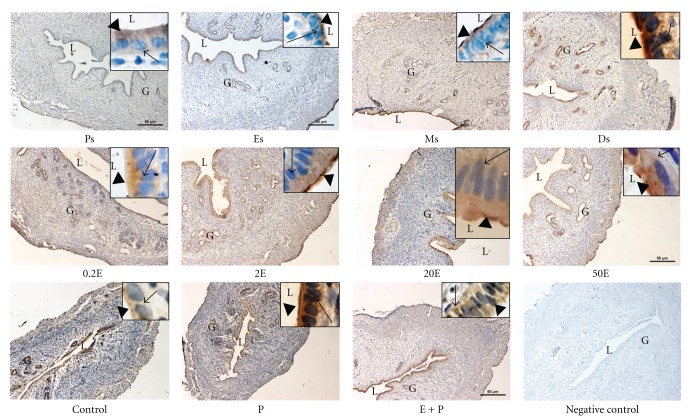
Immunolocalization of NHE1 in steroid replaced ovariectomized rats and in rats at different phases of the oestrous cycle. NHE1 expression was the highest under P dominance predominantly at the apical membrane. Magnifications of 10X and 100X (in the upper right corner). 0.2E: 0.2 **μ**g 17*β*-oestradiol, 2E: 2 **μ**g 17*β*-oestradiol, 20E: 20 **μ**g 17*β*-oestradiol, 50E: 50 **μ**g 17*β*-oestradiol, P: progesterone, E + P: 0.2 **μ**g 17*β*-oestradiol + progesterone. Ps: proestrus, Es: estrus, Ms: metestrus, Ds: diestrus. *n* = 6 rats per group. Incubation with a non-immune peptide was used as a negative control to check for the specificity of the antibodies. No staining was observed in this experiment.

In Figure 4 and Table 1, IHC results showed that NHE-1 was distributed mainly at the apical membrane of the luminal and glandular epithelia following sex steroid treatment. Minimal staining could be seen at the apical membrane in the 0.2E and 2E groups, while a moderate to strong staining predominantly at the apical membrane was observed in 20E group. In 50E group however, intense apical staining could be seen. In contrast to E, P treatment resulted in intense expression of NHE-1 predominantly at the apical membrane of the luminal and glandular epithelia. In the E + P group, however, no staining was observed. Meanwhile, throughout the oestrous cycle, NHE-1 was minimally expressed at Ps and Es mainly at the apical membrane. At Ms, a faint to moderate staining could be seen at the apical membrane while an intense staining; predominantly apical was observed at Ds. The intensity of NHE-1 expression in IHC following sex steroids treatment and at different oestrous cycle phases correlates with the pattern of NHE-1 mRNA and protein expression by real-time PCR and western blotting, respectively.

**Figure 5:**
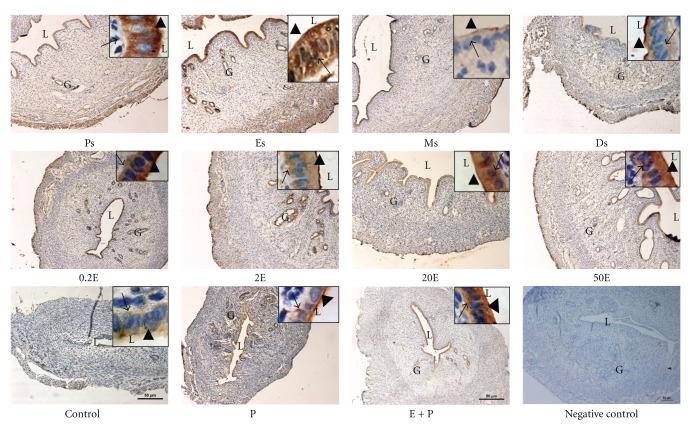
Immunolocalization of NHE2 in steroid replaced ovariectomized rats and in rats at different phases of the oestrous cycle. NHE2 was expressed at both the apical and basolateral membrane of the endometrial epithelia. Its expression was increased under E-dominance, however was reduced under P dominance. Magnifications of 10X and 100X (in the upper right corner). 0.2E: 0.2 *μ*g 17*β*-oestradiol, 2E: 2 *μ*g 17*β*-oestradiol, 20E: 20 *μ*g 17*β*-oestradiol, 50E: 50 *μ*g 17*β*-oestradiol, P: progesterone, E + P: 0.2 *μ*g 17*β*-oestradiol + progesterone. Ps: proestrus, Es: estrus, Ms: metestrus, Ds: diestrus. *n* = 6 rats per group. Incubation with a non-immune peptide was used as a negative control to check for the specificity of the antibodies. No staining was observed in this experiment.

In Figure 5 and Table 2, IHC findings showed that NHE-2 was expressed mainly at the luminal and glandular epithelia following sex steroids treatment and at oestrous cycle phases. Additionally, stromal and myometrial expression could also be seen at Ps and Es phases. Treatment with E resulted in a dose-dependent increase in the intensity of staining at both the apical and basolateral membranes of the luminal epithelia. The staining was the most intense following treatment with 20E and 50E. In the P-treated group however, minimal apical staining could be seen, while moderate to intense staining was observed in the E + P group. The distribution of NHE-2 throughout the oestrous cycle phases correlates with the changing level of sex steroid. The expression was the most intense at Ps and Es, while minimal to moderate expression could be seen at Ms and Ds.

**Figure 6:**
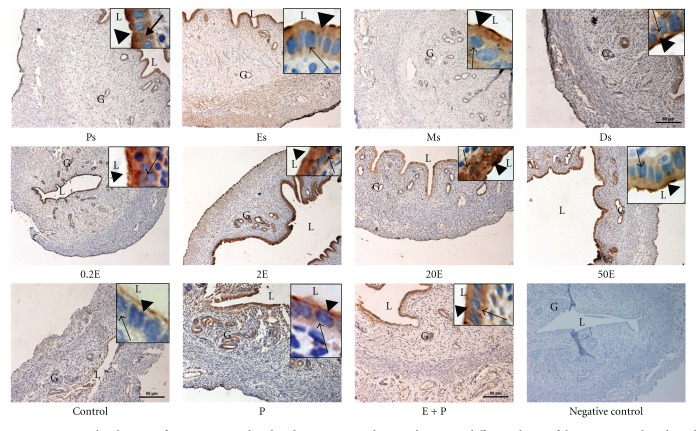
Immunolocalization of NHE4 in steroid replaced ovariectomized rats and in rats at different phases of the oestrous cycle. Bilateral distribution of NHE4 was observed following E treatment and at Ps and Es stages of the cycle, however the expression was reduced under P dominance. Magnifications of 10X and 100X (in the upper right corner). 0.2E: 0.2 *μ*g 17*β*-oestradiol, 2E: 2 *μ*g 17*β*-oestradiol, 20E: 20 *μ*g 17*β*-oestradiol, 50E: 50 *μ*g 17*β*-oestradiol, P: progesterone, E + P: 0.2 *μ*g 17*β*-oestradiol + progesterone. Ps: proestrus, Es: estrus, Ms: metestrus, Ds: diestrus. *n* = 6 rats per group. Incubation with a non-immune peptide was used as a negative control to check for the specificity of the antibodies. No staining was observed in this experiment.

In Figure 6 and Table 3, NHE-4 could be seen to be distributed at the apical and basolateral membrane of the luminal and glandular epithelia. The most intense staining was observed following treatment with low to moderate doses of E (0.2E, 2E, and 20E). Treatment with 50E and E + P resulted in a moderate increase in the staining intensity. Meanwhile, P treatment resulted in a minimal to moderate expression of NHE-4 at the apical membrane while no staining was observed in the control group. Throughout the oestrous cycle phases, however, the staining was the most intense at Ps and Es predominantly at the apical membrane, while only minimal to moderate staining could be seen at Ds.

In general, the intensity of NHE-2 and NHE-4 proteins in IHC following sex steroids treatment and at different phases of the oestrous cycle correlates with the observed changes in their mRNA and protein expressions by real-time PCR and western blotting, respectively.

## 4. Discussion

Despite the reported existence of NHE-1, -2, and -4 mRNAs in the endometrial epithelia from immature mice uteri [5], the effect of sex steroid and oestrous cycle phases on their expression remained largely unknown. To the best of our knowledge, this study is the first to reveal the following: (i) E upregulates the expression of NHE-4 and NHE-2 mRNAs and proteins while P causes a concomitant increase in NHE-1 mRNA and protein expression in the uterus, (ii) throughout the oestrous cycle, an increase in NHE-1 mRNA and protein expression occur at Ds while an increase in NHE-2 and NHE-4 mRNAs and proteins expression occur at Ps and Es, (iii) NHE-4 is the most abundant NHE isoform expressed in the uterus under E stimulation, and (iv) NHE-2 and NHE-4 are expressed at both the apical and basolateral membranes while NHE-1 is predominantly expressed at the apical membrane of the luminal and glandular epithelia.

Differential expression of NHE isoforms under sex steroid influence may explain the uterine fluid pH fluctuation at different phases of the menstrual/oestrous cycle. During the follicular phase and at around the time of ovulation (when E level is high), uterine luminal fluid has been reported to be alkaline due to an increase in HCO_3_^−^ content [9]. Under E effect, luminal HCO_3_^−^ secretion occurs via the cystic fibrosis transmembrane regulator (CFTR) [14, 15] and Cl^−^/HCO_3_^−^ exchanger (SLC26A6) [14, 16]. HCO_3_^−^ can either be generated intracellularly or derived from the plasma. Intracellular HCO_3_^−^ generation occur via the conversion of H_2_CO_3_ to H^+^ and HCO_3_^−^ by carbonic anhydrase (CA) [17]. Our unpublished observation indicates that E stimulates CA expression; therefore, E may indirectly stimulate intracellular HCO_3_^−^ generation. We have shown that under E stimulation, an increase in the expression of basolaterally located NHE-2 and NHE-4 may mediate intracellular H^+^ extrusion in exchange with Na^+^. This is important in order to maintain the intracellular pH homeostasis associated with a continuous luminal HCO_3_^−^ secretion [18]. Intracellular Na^+^ is generally believed to be pumped out via the basolaterally located Na^+^/K^+^-ATPase [19].

In addition to an increase in the basolateral expression, we have also shown that E upregulates the expression of NHE-2 and NHE-4 at the apical membrane of the luminal epithelia. The role of apical NHE-2 and NHE-4 is unknown; however, these isoforms may participate in luminal Na^+^ secretion, which has been shown to be enhanced under E influence [20]. It has long been established that E stimulates fluid and electrolytes secretion in the endometrium [21]. Na^+^ secretion has been thought to occur exclusively via a leaky tight junction [22] since no transporters responsible for this process has been identified at the apical membrane of the endometrium. Aronson et al. [23] demonstrated that Na^+^ efflux can be stimulated by intracellular H^+^. Other findings in a native or NHE-transfected cells suggested that NHE exchange activity is dependent on internal H^+^ rather than external Na^+^ or H^+^ concentrations [24]. Wakabayashi et al. [25] reported that intracellular pH- (pHi-) dependent Na^+^ efflux from cells expressing NHE-1, NHE-2, and NHE-3 was stimulated by intracellular acidification and was completely inhibited by intracellular alkalinization. These observations support our hypothesis that under E dominance, apical NHE-2 and NHE-4 may catalyze a reverse mode of exchange of intracellular Na^+^ for extracellular H^+^, thus promoting Na^+^ secretion into the uterine lumen. In addition to HCO_3_^−^ secretion via the CFTR [15, 26] and SLC26A6 [14, 16], which were known to be upregulated by E, H^+^ reabsorption will further increase the pH of the uterine fluid. Meanwhile, E has also been reported to down-regulate the activity of basolaterally located Na^+^/K^+^-ATPase [27], which may further promote the luminal Na^+^ secretion.

**Figure 7:**
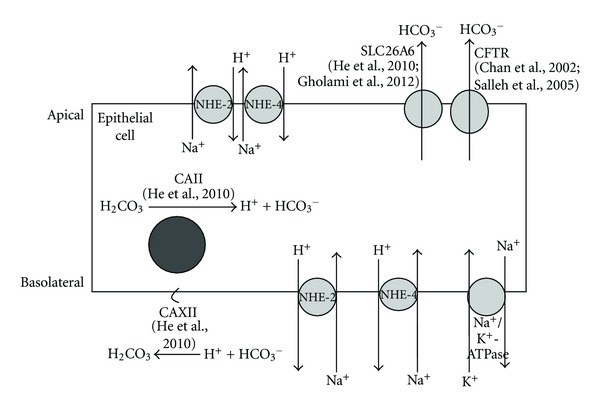
The proposed mechanism underlying uterine fluid pH regulation in the endometrial epithelial cell under E influence and at Es. CAII, which is responsible for H^+^ and HCO_3_^−^ generation, is upregulated by E and at Es. HCO_3_^−^ is extruded into the lumen via the apically located SLC26A6 and CFTR, which have also been reported to be upregulated by E and at Es. In order to maintain intracellular pH homeostasis, H^+^ is expelled into the plasma via the basolaterally located NHE2 and NHE4 in-exchange with Na^+^, which is pumped back into the plasma via the basolaterally located Na^+^/K^+^-ATPase. The apically located NHE2 and NHE4 may be involved in luminal Na^+^ secretion in-exchange with H^+^, which further contributes to the alkalinization of the uterine luminal fluid via H^+^ removal. Upregulation of membrane bound CAXII under these conditions is responsible for the conversion of H^+^ to H2CO3. CAII: carbonic anhydrase II; CAXII: carbonic anhydrase XII; CFTR: Cystic Fibrosis Transmembrane Regulator, SLC26A6: Cl^−^/HCO_3_^−^ exchanger, NHE: sodium-proton exchanger, Na^+^/K^+^-ATPase: Na^+^-K^+^-ATPase pump.

**Figure 8:**
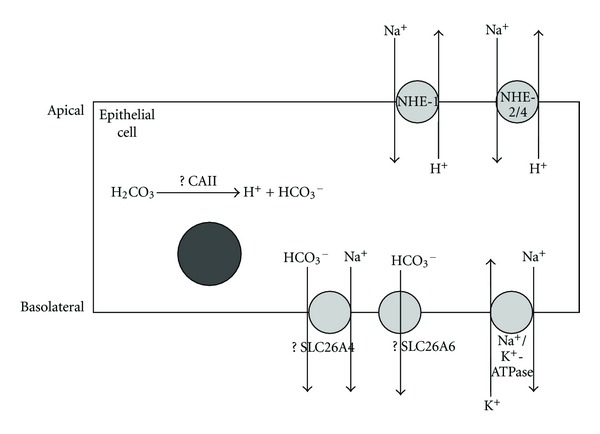
The proposed mechanism underlying uterine fluid pH regulation in the endometrial epithelial cell under P influence and at Ds. CAII may be involved in intracellular generation of H^+^ and HCO_3_^−^. H^+^ will then be extruded into the lumen via the apically located NHE1. NHE2 and NHE4, which are expressed at the apical membrane at a lower level than NHE1 may also participate in luminal H^+^ secretion in-exchange with Na^+^. Meanwhile, HCO_3_^−^ is expelled into the plasma via the basolaterally located SLC26A6 or other HCO_3_^−^ transporters including SLC24A4 (although their expression at the basolateral membrane under P influence is unknown) to maintain the intracellular pH homeostasis. Na^+^ that accumulates in the cell will be extruded into the plasma via basolateral Na^+^/K^+^-ATPase or SLC24A4. The mechanism underlying uterine fluid pH regulation under these conditions is not well understood as compared to the mechanisms involved under E dominance. CAII: carbonic anhydrase II, SLC26A6: Cl^−^/HCO_3_^−^ exchanger, SLC24A4: sodium bicarbonate cotransporter, NHE: sodium-proton exchanger, Na^+^/K^+^-ATPase: Na^+^-K^+^-ATPase pump.

We have also shown that NHE-1 mRNA and protein expressions were upregulated by P and at Ds stage of the cycle. NHE-1 was predominantly found at the apical membrane supporting the view that this isoform participates in intraluminal fluid acidification under P influence (our unpublished data). Additionally, the apically located NHE-1 could also mediate Na^+^ reabsorption in exchange with H^+^. Typically, Na^+^ reabsorption has been reported to occur via the epithelial Na^+^ channel (ENaC) [26], however in tissues lacking ENaC such as epididymis, Na^+^ reabsorption may also occur via the NHE [8]. NHE-mediated Na^+^ reabsorption and H^+^ secretion has been reported in the kidney, pancreas, intestine, colon, stomach, biliary tree, and middle ear [28–35]. In human and mouse choroid plexuses, luminal NHE-1 mediates net proton secretion into the cerebrospinal fluid (CSF) [36]. In addition to luminal fluid acidification, apical NHE has also been reported to participate in luminal fluid absorption driven by hypertonicity of the paracellular fluid secondary to Na^+^ reabsorption. The role of NHE in luminal fluid loss has been described in the epididymis, kidney proximal tubules, and intestinal epithelia [37, 38]. An increase in NHE-1 expression at the apical membrane of the luminal epithelia could be responsible for the uterine luminal fluid acidification and secondary fluid loss observed under P influence. Fluid loss may assist uterine closure, which brought the blastocyst in contact with the two opposing surfaces of the endometrium [39]. The significance of P-mediated increased in H^+^ secretion is unknown; however, it may help in creating an acidic environment required for blastocyst implantation [40]. An increased in uterine fluid H^+^ concentration may help to reduce the electrostatic repulsion force between the negatively charged endometrium and the blastocyst in addition to the blastocyst itself losing part of its negative charge at the time of implantation [41]. As mentioned earlier, the intracellular proton generation may occur via the action of CA. In order to maintain the intracellular pH homeostasis, HCO_3_^−^ is extruded into the plasma. The mechanisms underlying parallel HCO_3_^−^ extrusion via the basolateral membrane under P were not known, however it may involve the HCO_3_^−^ transporters such as SLC26A6 [14, 16] and SLC26A4 [42]. In addition, there is a possibility that P may also increase the expression and activity of endometrial Na^+^/K^+^-ATPase, which promotes luminal Na^+^ reabsorption although there is no current evidence to support this. The proposed mechanisms underlying the effects of sex-steroid on uterine fluid pH regulation are summarized in Figures 7 and 8.

In conclusion, differential expression of NHE isoforms under the effect of sex steroid and throughout the oestrous cycle may have important implication towards the normal regulation of the uterine fluid pH and volume. Under E dominance, NHE-2 and NHE-4 upregulation may assist uterine fluid alkalinization and intraluminal Na^+^ secretion. Under P dominance, however, NHE-1 upregulation may mediate intraluminal H^+^ secretion in exchange with Na^+^, thus facilitating secondary fluid loss which contributes to uterine closure that initiate the attachment phase of embryo implantation.

## Figures and Tables

**Table 1:** Semiquantitative assessment on the intensity of the staining of NHE-1.

	Apical	Basolateral
control	−	−/+
0.2E	−	−/+
2E	−	+
20E	++	+
50E	+++	+
P	+++	+
E + P	−	−
Ps	−/+	−
Es	−/+	−
Ms	−/+	−
Ds	+++	+

Semi-quantitative assessment on the intensity of the staining of NHE1. The staining intensity was graded as the following: −: negative; −/+: faint; +: moderate; ++: strong; and +++: very intense, as described previously [13]. The intensity was confirmed by 3 independent observers. 0.2E: 0.2 *μ*g 17*β*-oestradiol, 2E: 2 *μ*g 17*β*-oestradiol, 20E: 20 *μ*g 17*β*-oestradiol, 50E: 50 *μ*g 17*β*-oestradiol, P: progesterone, E + P: 0.2 *μ*g 17*β*-oestradiol + progesterone. Ps: proestrus, Es: estrus, Ms: metestrus, Ds: diestrus.

**Table 2:** Semiquantitative assessment on the intensity of the staining of NHE-2.

	Apical	Basolateral
control	−/+	−
0.2E	++	+
2E	++	++
20E	+++	++
50E	+++	++
P	+	−
E + P	+++	++
Ps	+++	+++
Es	+++	+++
Ms	−/+	−
Ds	+	−/+

Semi-quantitative assessment on the intensity of the staining of NHE2. The staining intensity was graded as the following: −: negative; −/+: faint; +: moderate; ++: strong; and +++: very intense, as described previously [13]. The intensity was confirmed by 3 independent observers. 0.2E: 0.2 *μ*g 17*β*-oestradiol, 2E: 2 *μ*g 17*β*-oestradiol, 20E: 20 *μ*g 17*β*-oestradiol, 50E: 50 *μ*g 17*β*-oestradiol, P: progesterone, E + P: 0.2 *μ*g 17*β*-oestradiol + progesterone. Ps: proestrus, Es: estrus, Ms: metestrus, Ds: diestrus.

**Table 3:** Semiquantitative assessment on the intensity of the staining of NHE-4.

	Apical	Basolateral
control	−/+	−
0.2E	+++	+++
2E	+++	++
20E	+++	+++
50E	+	−/+
P	+	−/+
E + P	++	++
Ps	+++	++
Es	+++	+
Ms	+++	−/+
Ds	+	−/+

Semi-quantitative assessment on the intensity of the staining of NHE4. The staining intensity was graded as the following: −: negative; −/+: faint; +: moderate; ++: strong; and +++: very intense, as described previously [13]. The intensity was confirmed by 3 independent observers. 0.2E: 0.2 *μ*g 17*β*-oestradiol, 2E: 2 *μ*g 17*β*-oestradiol, 20E: 20 *μ*g 17*β*-oestradiol, 50E: 50 *μ*g 17*β*-oestradiol, P: progesterone, E + P: 0.2 *μ*g 17*β*-oestradiol + progesterone. Ps: proestrus, Es: estrus, Ms: metestrus, Ds: diestrus.
